# Presence and distribution of mosquito larvae predators and factors influencing their abundance along the Mara River, Kenya and Tanzania

**DOI:** 10.1186/s40064-015-0905-y

**Published:** 2015-03-20

**Authors:** Gabriel O Dida, Frank B Gelder, Douglas N Anyona, Paul O Abuom, Jackson O Onyuka, Ally-Said Matano, Samson O Adoka, Canisius K Kanangire, Philip O Owuor, Collins Ouma, Ayub VO Ofulla

**Affiliations:** School of Public Health and Community Development, Maseno University, Kisumu, Kenya; Department of Vector Ecology and Environment, Institute of Tropical Medicine (NEKKEN), Nagasaki University, Nagasaki, Japan; Probe International, Inc., USA and Auckland, Ohio, New Zealand; School of Environment and Earth Science, Maseno University, Kisumu, Kenya; EAC-Lake Victoria Basin Commission Secretariat, Kisumu, Kenya; Department of Chemistry, Maseno University, Kisumu, Kenya

**Keywords:** Coleoptera, Fish, Hemiptera, Mara river, Mosquito larvae, Odonata, Predators

## Abstract

Among all the malaria controlling measures, biological control of mosquito larvae may be the cheapest and easiest to implement. This study investigated baseline predation of immature mosquitoes by macroinvertebrate predators along the Mara River, determined the diversity of predators and mosquito larvae habitats and the range of their adaptive capacity to water physico-chemical parameters. Between July and August 2011, sampling sites (n=39) along the Mara River were selected and investigated for the presence of macroinvertebrate predators and mosquito larvae. The selected sampling sites were geocoded and each dipped 20 times using standard mosquito larvae dipper to sample mosquito larvae, while a D-frame dip net was used to capture the macroinvertebrate predators. Water physico-chemical parameters (dissolved oxygen, temperature, pH, conductivity, salinity and turbidity) were taken *in situ* at access points, while hardness and alkalinity were measured titrimetically. The influence of macroinvertebrate predator occurrence was correlated with mosquito larvae and water quality parameters using Generalized Linear Model (GLM). Predators (n=297) belonging to 3 orders of Hemiptera (54.2%), Odonata (22.9%) and Coleoptera (22.9%), and mosquito larvae (n=4001) belonging to 10 species, which included *An.gambiae* s.l (44.9%), *Culex* spp. (34.8%) and *An. coustani* complex (13.8%), *An. maculipalpis* (3.6%), *An. phaorensis* (1.2%), *An. funestus* group (0.5%), *An. azaniae* (0.4%), *An. hamoni* (0.3%), *An. christyi* (0.3%), *An. ardensis* (0.08%), *An. faini* (0.07%), *An. sergentii* (0.05%) and 0.05% of Aedes mosquito larvae which were not identified to species level, due to lack of an appropriate key, were captured from different habitats along the Mara river. It was established that invasion of habitats by the macroinvertebrate predators were partially driven by the presence of mosquito larvae (*p* < 0.001), and the prevailing water physico-chemical parameters (DO, temperature, and turbidity, *p* <0.001). Understanding abiotic and biotic factors which favour mosquitoes and macroinveterbrate co-occurrence may contribute to the control of malaria.

## Introduction

Like in many other parts of the sub-Saharan Africa, malaria is increasingly becoming a major health problem among communities living within river basins including the Mara River basin, which stretches between the Maasai Mara game reserve in Kenya and Serengeti National Park in Tanzania (Bussman et al. 2006). Malaria is now the leading cause of morbidity and mortality among children in many districts within the Lake Victoria basin, such as Trans Mara District, which falls within the Mara River basin of Kenya, while in Tanzania, the disease is common in almost all regions; the Maasai Mara game reserve being classified as low to moderate malaria epidemic area in East Africa (Schlagenhauf-lawlor and Scott 2001). According to the Serengeti Mara Camp Fact sheet, 2013, the famous Serengeti National Park in Tanzania also falls within a malaria endemic zone.

One of the most common strategies to eradicate malaria has always focused on mosquito control by use of various chemicals including insecticides. However, their use have had a negative impact on non-target organisms and the environment. Studies have also shown that some of the chemicals used kill natural mosquito predators more effectively than the target mosquitoes and over time, predators such as fish and insects die out while mosquitoes develop resistance, multiplying in ever larger numbers in a losing battle often referred to as “the pesticide treadmill” (Wilson and Tisdell 2001). Moreover, the application of insecticide strategies has also failed due to the development of insecticide resistance and lack of knowledge about the behavior of the vectors (Shiff 2002, Kawada et al. 2011, 2014). The non-selective nature and use of pesticides therefore leaves biological control of mosquito larvae as among the best and most environmentally-friendly option for the control of mosquitoes.

The role of predatory aquatic insects in the natural regulation of mosquito larvae has been reported by many researchers (Knight et al. 2003; Tuno et al. 2005; Mogi 2007; Quiroz-Martinez and Rodriguez-Castro 2007; Shaalan et al. 2007). However, predators vary markedly in the different habitats that immature and adult mosquito frequent. Representatives from at least six insect orders, thirteen arachnid families, as well as crustaceans, amphibians, fish, birds and mammals have been reported as being potential mosquito larvae predators (Mogi 2007, Medlock and Snow, 2008). Some studies in Kenya reported mosquito larva predation in rice irrigation schemes and wetlands around Lake Victoria (Mwangangi et al. 2007; Minakawa et al. 2007; Ohba et al. 2010). However, most of these studies limit their research to specific types of aquatic insects, principally the Family Notonectidae (Koivisto et al. 1997; Murdoch et al. 1984). In western Kenya, especially around Lake Victoria, members of the *Anopheles gambiae* s.l. and *An. funestus* dominate (Minakawa et al. 2008; Minakawa et al. 2012; Kweka et al. 2013).

Past experimental studies confirmed that predation on immature mosquitoes by macroinvertebrates can be a major driving force in controlling the population size of mosquitoes, especially the malaria vectors. For instance, Chandler and Highton (1977), reported how predation on *An. gambiae* larvae resulted in the reduction of the population of the vectors considerably by between 13.4% and 84.5%, respectively. An overall larval mortality of between 92.6% and 97.1% was also reported by Service (1971), (1973) and (1977). Different fish species have also proved to be effective in mosquito larvae control. However, little research has focused on the assessment of the available predators’ local ecology to establish their impact on mosquito population. Several factors are, however, known to affect the predator-prey relationship. They include preference or selectivity of the prey by the predator, species diversity in mosquito breeding sites, stability of the aquatic system, larval density, position of the predator in the water column, and predator to prey ratio for the selected micro-environment. Predator-prey co-evolution, predator-prey synchronization and refuge are also important contributing factors (McPeek and Miller 1996). Environmental factors including temperature (Anderson et al. 2001), dissolved oxygen, conductivity (Spieles and Mitsch 2000), and pH (Adebote et al. 2008) may affect predator and prey numbers. From these studies it has been proposed that fluctuating abiotic conditions and interactions among species affect predators and prey differentially (Anderson et al. 2001). The physico-chemical differences between mosquito and predator breeding habitats are poorly understood and little effort has been made to understand how these factors affects the vector and prey population in a shared habitat.

The effective control of malaria through vector management requires information on the distribution and abundance of vectors, as well as factors that favour their adaptation in the targeted areas. Mosquito larval control is one potentially important target point in malaria vector control (Kumar et al*.*^2008^; Kweka et al. 2011). Understanding each species biological limits to abiotic factors as well as their habitat structure across environmental gradients may provide useful insight into how assemblages of mosquitoes and mosquito predators are structured. This information then become useful for proper application of biological control of mosquito larvae. Save for a few studies that have been carried out in the laboratory with species of *Anopheles* and *Culex* (Tuno et al. 2006, 2007)*,* little is known on the predation of mosquitoes from rivers and streams in Kenya.

This study was therefore designed to determine the presence and distribution of mosquito larvae predators along the Mara River basin, Kenya and Tanzania, for future planning of intervention strategies against malaria and other mosquito-borne diseases through biological control. An attempt was also made to further understand the role of water physico-chemical parameters on habitat stability. The mean range requirement of physico-chemical parameters in habitats shared by the mosquito larvae and predators under field/natural conditions was analyzed from the data collected within 2 months from 39 sampling sites along the Mara River. The identification of indigenous predator populations and their tolerance to environmental variables may help curtail the insurgent of the vector mosquitoes if a predator propagation program can be initiated.

## Materials and methods

### Study area

This cross-sectional study was conducted at the trans-boundary Mara River basin which lies between longitudes 33^0^47’E and 35^0^47’E and latitudes 0^0^38’S and 1^0^52’S, traversing Kenya and Tanzania, in East Africa (Mutie et al. 2006). The basin has a tropical rainforest climate with two distinct seasons; the rainy season occurring between March and May, and the dry season between June and October (Mati et al. 2005). Rainfall in the basin varies with height, ranging from 1,000 to 1,750 mm in the Mau Escarpment, to 900 and 1,000mm in the middle rangelands and 700 to 850mm in the lower Loita hills and around Musoma in Tanzania, where the river discharges into Lake Victoria. The dominant land uses within the basin are agriculture, pastoralism and wildlife sanctuaries (Mati et al. 2008).

### Sampling design

Sample collection was carried out from the beginning of July 2011 to end of August 2011 (dry season) from 39 sampling sites along the Mara River. This study period was selected as it presents some of the most extreme environmental variables throughout the year. The specific sampling points were chosen within a 100m interval from one end of the river bank to the other. The sites were coded based on their location and point of sampling. These points were at times strategically chosen before and after a bridge or a through road (for ease of access of both sides of the river), and thus the sampling sites on either side of the bridge or road were labeled systematically with the first letters denoting their location as either being upstream or downstream part of the river, while taking the bridge as the reference point (i.e. URS1-10 and DRS1-10). For instance, URS 1-10 denoted that the sampling sites 1 to 10 were located on the upstream side of the river or its tributaries before a bridge, while DRS 1-10 were located on the downstream side after the bridge. Other habitats adjacent were given Latin numbers with their nature and/or name of the habitat described in detail. This kind of labeling was done to avoid any confusion and for easier analysis of the specimen. The geographical locations of all the sampling sites were taken at access points and recorded as GPS co-ordinates. At every site, all probable habitat types found were recorded, classified and inspected for the presence or absence of mosquito larvae and their predators. Each habitat was dipped 20 times using a standard mosquito dipper (350 ml; BioQuip Products, Rancho Dominguez, USA). A D-frame dip net of 0.3m width attached to a long pole and with a cone-shaped bag for capturing the mosquito larvae predators was used. The sampling was done from upstream to downstream end of the river. A total of 3 collections were made at each sampling point with the collection consisting of a forceful thrust of the sampler into the sediment for a linear distance of 0.5m. The captured mosquito larvae and predators were immediately preserved in 90% ethanol for further identification. Water physico-chemical parameters (DO, pH, temperature, conductivity, turbidity and salinity) were measured *in situ* by use of a hand-held multi-parameter-YSI meter (YSI Model 650-01m Environmental Monitoring Systems, Yellow Springs, OH), while hardness and alkalinity were measured titrimetically.

An electro-fisher was used to sample fish only from the Mara River tributaries of Amala and Nyangores tributaries and the main Mara River. Five sites were chosen randomly and their coordinates taken to represent sampling points in both Kenya and Tanzania. Fish samples were obtained as per methods described by Matano (2013). Briefly, an electro-fisher that uses a pulsed current was used for fishing. This tool was connected to an external generator that powered it to produce electric current. The fishing duration at each station lasted approximately 30 minutes and covered a distance of 50m and a width of about 3m. Fish sampled were identified to species level using morphometric and meristic characteristics following descriptions given by Witte and Van Oijen (1990) and Greenwood (1981). Water physico-chemical parameters were also taken to establish their influence on fish abundance. Data from these sites were however not included in the final model for the macroinvertebrates and mosquito analysis as they failed to represent all sites and the procedure for sampling was also non-selective.

### Sampling points

Sampling points (n = 39) were surveyed along the Mara River and its tributaries. The sampling points comprised of macro-habitats including: river (n = 10), drying stream (n = 10), swamps (n = 8), open puddles (n = 5), rock pools (n = 6), dam sites (n = 4), hoof prints (n = 12), vegetated pools (n = 17) and drainages (n = 25). The remaining sites (n = 29) were mainly open sun-lit pools such as brick-making sites and drainages associated with agricultural activities in the ephemeral habitats adjacent to the river.

### Laboratory analysis and identification of mosquito larvae and the predators

All the collected mosquito larvae were identified microscopically using standard taxonomic methods (Gillies and Coetzee 1987). During the sampling process, the relatively large macroinvertebrate predators such as *Anisops wakefieldi* (back-swimmers), Rhantus larvae (diving beetles) and *Onychohydrus hookeri* (water beetles) were visually observed, classified and counted. Those that could not be identified to the species level in the field, including fish were preserved for further identification using appropriate keys as described by Jenkins (1964), Merritt and Cummins (1996), Nilson (1996, 1997), Verschuren (1997), Anderson et al. (2001), and by use of lists of species commonly present in Kenya as described by Johanson (1992) and Mathooko (1998). The number of mosquito larvae predators was recorded for each sampled habitat.

## Statistical analysis

The mean differences in water physico-chemical parameters per habitat types were compared using One-way ANOVA, while the relationship between predators and mosquito larval abundance and the water physico-chemical parameters was determined using the generalized linear model with negative binomial in MASS package, and log as defunct link function. In the initial steps of the analysis, all the variables were first explored for their distribution and the homogeneity of variance checked using histograms and dot charts after which the most appropriate link function was chosen. The initial model was built around the premise that the distribution of the response variable was Poisson, whose over-dispersion was evaluated, and when the conditional variance exceeded the conditional mean, a generalized linear model with negative binomial was employed. It is considered as a generalization of Poisson regression since it has the same mean structure as Poisson regression and has an extra parameter to model the over-dispersion. A full model included all relevant covariates, which was then simplified until the best model with smallest Akaike Information Criterion (AIC) obtained following the stepwise removal of the covariates. The final model was built as follows; formula=(Predators’ abundance) ~ Total mosquito larvae + Dissolved oxygen + Temperature + Turbidity + pH, (family=GLM.nb). The mean range of water physico-chemical parameters requirements by both mosquito larvae and their predators in the same habitats were evaluated using Canonical Correlation Analysis (CCA), as described by Knapp (1978), Härdle and Simar (2007) and Skourkeas et al. (2010), while the contribution of each variable in the shared habitats was determined using Ordination Analysis (OA). Reliability coefficient of the physico-chemical parameters on fish abundance was determined using Pearson’s Rank Order Correlation Analysis. All statistical analyses were performed using R (R Core Team, 2013). An alpha value (*p <* 0.05) was considered statistically significant.

## Results

**Figure 1:**
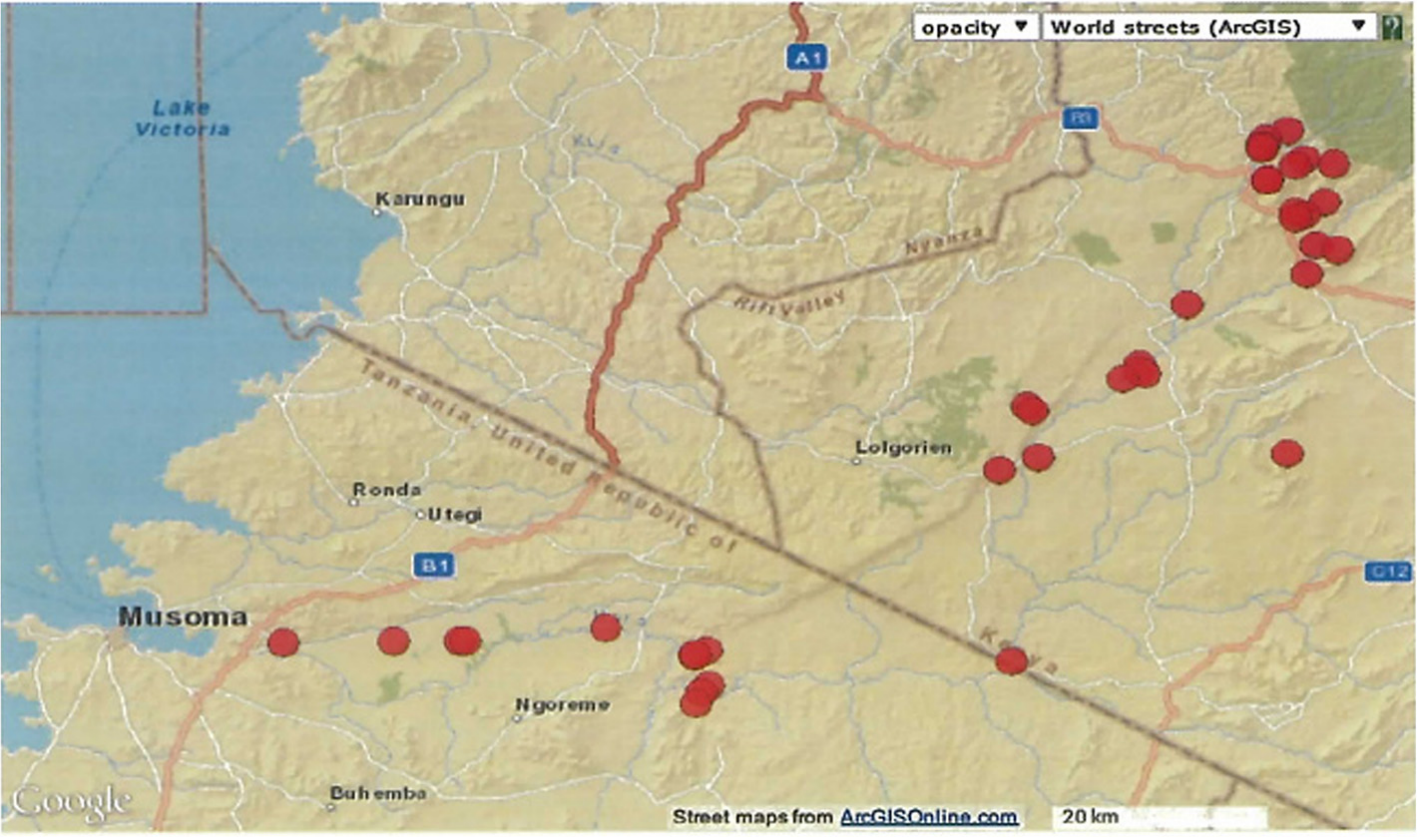
**Red dots show the sampling sites along the Mara river and tributaries, Kenya and Tanzania (n = 39).**

**Figure 2:**
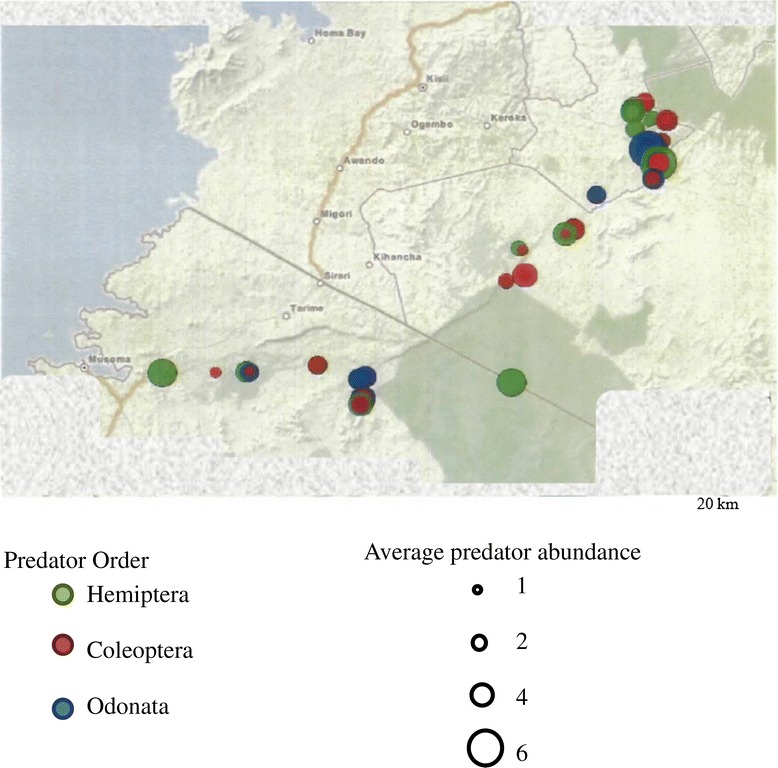
**Dot size and color show predator order and average number at sampling sites along the Mara river and tributaries, Kenya and Tanzania (n = 39).**

**Figure 3:**
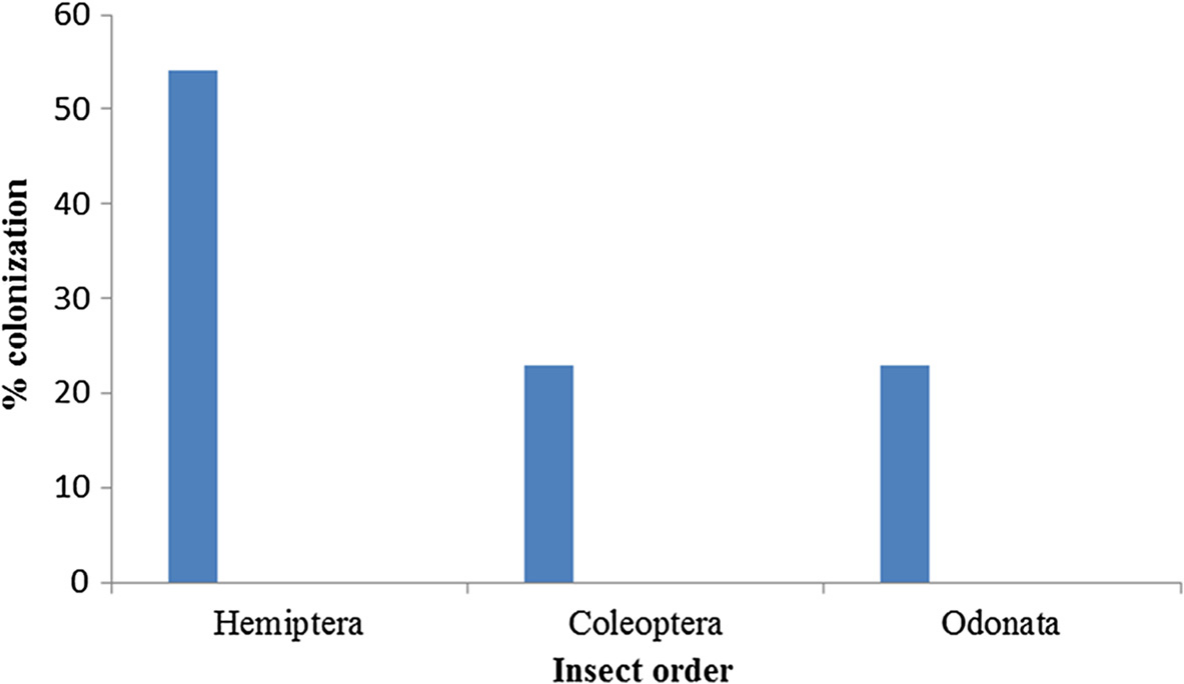
**Abundance of macoinvertebrate predators by order along the Mara river and its tributaries, Kenya and Tanzania, n = 39.**

Sampling sites and predator distribution along the Mara River and its tributaries are shown in Figure 1 and Figure 2, respectively. A total of 297 macroinvertebrate predators belonging to 3 orders–Hemiptera (54.2%), Odonata (22.9%) and Coleoptera (22.9%) were collected (Table 1). Seven families were recorded within the Order Hemiptera, with members of Family Velidae and genus *Rhagovelia* dominating. Three families were registered within Odonata, dominated by Family Coenagrionidae, while Order Coleoptera had 2 families dominated by Dytiscidae (Table 1). In addition, a total of 4001 mosquito larvae were recorded belonging to 10 species, which included *An.gambiae* s.l (44.9%), *Culex* spp. (34.8%) and *An. coustani* complex (13.8%), *An. maculipalpis* (3.6%), *An. pha*o*roensis* (1.2%), *An. funestus* group (0.5%), *An. azaniae* (0.4%), *An. hamoni* (0.3%), *An. christyi* (0.3%), *An. ardensis* (0.08%), *An. faini* (0.07%), *An. sergentii* (0.05%) and 0.05% of Aedes mosquitoes which were not identified to species level due to lack of an appropriate key. The mosquito larvae were mainly collected in drying stream, swamps vegetated puddles and open water pools. The majority were collected in drying stream where predators were also dominant. The macroinvertebrate predators from three genera were more abundant where mosquito larvae were present (Table 2). The distribution of predators and mosquito larvae per habitat type is as presented in Figure 3. Mosquitoes were captured in the following habitats: drying stream (40.1%), swamps (20.0%), vegetated pools (16.4%), dam (12.4%), open puddles (7.8%), livestock hoof-prints (2.0%) and rock pools (1.3%), among others.

**Table 1:** **Order, family, genus, number and percent (%) for all of mosquito larvae predators captured throughout this study at all locations**

**Order (n)**	**Family (common name)**	**Genus/species (sub-order)**	**n (%)**
Hemiptera (161)	Gerridae	Hynesionella (Nepomorpha)	7 (2.4)
		Limnogonus (Gerromorpha)	13 (4.4)
	Hydrometridae	Hydrometra species	15 (5.1)
	Veliidae	Rhagovelia (Heteroptera)	38 (12.8)
	Notonectidae	Anisops (Anisoptera)	30 (10.1)
		Enithares species	9 (3.0)
	Pleidae (Water bug)	Pleidae species	8 (2.7)
	Naucoridae	Naucoridae species	7 (2.4)
	Nepidae	Ranatra species	10 (3.4)
		Laccotrephes	24 (8.1)
Odonata (68)	Lestidae (Damselfly)	Lestes species	20 (6.7)
	Coenagrionidae	Enallagma species	21 (7.0)
	Libellulidae	Palpopleura	14 (4.7)
		Orthetrum albistylum	13 (4.4)
Coleoptera (68)	Hydrophilidae (Water beetle)	Hydrochara caraboides	8 (2.7)
	Dytiscidae	Laccophillus species	49 (16.7)
		Copelatus species	4 (1.3)
		Cybister species	6 (2.0)
		Hydaticus species	1 (0.3)
TOTAL (N)			297 (100)

**Table 2:** **Mosquito larvae and predator numbers in different habitats within the Mara river basin**

**Habitat**	**Mosquito (n)**	**Proportion (%)**	**Predators (n)**	**Proportion (%)**
Drying stream	1009	25.2	120	40.4
Swamps	830	20.7	92	31.0
Open puddles	524	13.1	4	1.4
Dams	510	12.8	13	4.4
Vegetated pools	455	11.4	45	15.2
Hoof prints	250	6.3	4	1.4
Drainages	234	5.8	13	4.4
Rock pools	188	4.7	3	1.0
River	1	0.0	3	1.0
TOTAL (N)	4001	100	297	100

**Figure 4:**
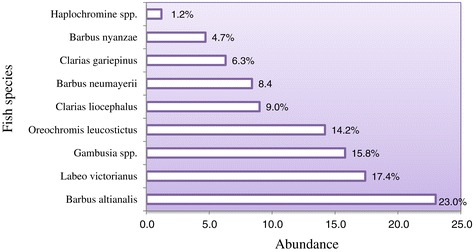
**Fish species sampled in the Mara river and its tributaries, n = 5.**

Nine species of fish (n=140) representing 4 families (Cyprinidae, Cichlidae, Claridae, and Poecillidae) were captured and identified in the five sampling sites, two on the main river and three on its tributaries. Cyprinids were the most abundant, with *Barbus altianalis* and *Labeo victorianus* being the most dominant (Figure 4). The most widely distributed species were *Barbus altianalis* and *Gambusia* spp. occurring in all sampling sites. These were followed by *Labeo victorianus* that was present in two sampled sites. Overall, the majority of the fish were caught on the main Mara River (60.9%) as compared to the Mara River tributaries; Nyangaores (20.3%) and Amala (18.8%).

Except for hardness and salinity, correlations between six other physico-chemical variables and fish abundance were evident at all the five sites but the number and strength of correlations clustered in distinct sites. For instance, the strength (significance level) of associations was greater at upstream sites. Dissolved oxygen and temperature correlated strongly with fish abundance at sites 1, 2 and 3 whereas sites 4 and 5 although characterized by relatively swift flow rate, fewer significant associations were recorded (Table 3).

**Table 3:** **Spearman rank order correlation results for association between fish abundance and the physico-chemical parameters at the Mara River and tributaries, n = 5**

**Variable**	**Site 1**	**Site 2**	**Site 3**	**Site 4**	**Site 5**
Dissolved Oxygen	0.65***	0.62***	0.44**	−	−
pH	0.52**	0.28*	−	−	−
Conductivity	0.24*	−	0.27*	−	−
Turbidity	0.38**	−	−	−	−
Temperature	0.66***	0.74***	−	0.18	0.38**
Hardness	−	−	−	−	−
Salinity	−	−	−	−	−

p <0.1, *p <0.05 **p <0.001 ***p <0.005; denotes strengths of correlation at different sites. Dissolved oxygen and temperature correlated strongly with fish abundance at sites 1, 2 and 3.

Along the Mara River, dissolved oxygen varied considerably among the breeding sites in which the macroinverterbrate predators and mosquito larvae were caught, with the highest DO recorded in the river (6.4 ± 0.7 mg/L), followed by rock pools (6.0 ± 0.7 mg/L). The lowest was recorded in swamps (2.4 ± 2.7 mg/L). The overall mean DO in puddles was (5.6 ± 0.8 mg/L), while that of drying stream was (5.3 ± 1.6 mg/L) (Table 4). A significant difference in mean DO was observed among the 9 different habitat types (ANOVA, n = 9, F = 4.2417, d.f. = 8, 26, *p* < 0.01). It was established that both mosquito larvae and macroinverterbrate predators along the Mara River were prevalent in samples with DO values ranging between 6.0 mg/L, to 6.5 mg/L.

**Table 4:** **Average physico-chemical parameters at different mosquito larvae habitats along Mara the River basin**

**Habitat**	**DO (mg/L)**	**pH**	**Alkalinity (mg/L)**	**Hardness (mg/L)**	**Turbidity (NTU)**	**Conductivity (μS/cm)**	**Temperature (** ^**0**^ **C)**	**Salinity (mg/L)**
Dams	4.7 ± 1.8	8.1 ± 0.4	100 ± 62.4	87.7 ± 56.2	96.9 ± 142.0	269.8 ± 213.8	24.4 ± 1.9	0.0 ± 0.0
Drying stream	5.3 ± 1.6	8.1 ± 0.6	126.2 ± 26.5	102.4 ± 68.9	124.3 ± 152.6	290 ± 186.5	22.5 ± 2.1	0.0 ± 0.0
Swamps	2.4 ± 2.7	7.0 ± 1.3	244.5 ± 274.6	58.5 ± 46.7	142.2 ± 108.5	174.3 ± 59.2	23.2 ± 4.9	<0.1
Drainages	4.3 ± 3.8	7.3 ± 0.5	400 ± 282.8	372 ± 393.2	144.8 ± 84.3	168.5 ± 13.4	24.2 ± 0.7	0.0 ± 0.0
Rock pools	6.0 ± 0.7	7.1 ± 0.8	153 ± 60.8	127 ± 69.3	542.6 ± 2.3*	368.0 ± 125.9*	26.2 ± 3.4	0.0 ± 0.0
Open puddles	5.6 ± 0.8	8.2 ± 0.5	104 ± 73.0	188 ± 247.7	95.2 ± 131.9	168.8 ± 87.3	25.2 ± 2.3	0.0 ± 0.0
River	6.4 ± 0.7	7.3 ± 0.4	100 ± 99.2	178 ± 228.8	135.2 ± 142.4	144.5 ± 97.6	19.7 ± 2.3	0.0 ± 0.0
Hoofprints	6.2 ± 0.5	8.1 ± 0.3	133 ± 50.2	98.9 ± 46.5	100.2 ± 62.1	140.3 ± 90.4	26.2 ± 1.9	0.0 ± 0.0
Vegetated pools	5.4 ± 0.6	8.0 ± 0.2	120 ± 72.5	104.1 ± 98.8	150.2 ± 102.4	135.2 ± 142.4	19.7 ± 2.3	0.0 ± 0.0

*Elevated levels of turbidity and conductivity were recorded in rock pools, probably due accumulation of dissolved particles.

Conductivity levels across different habitats showed wide variation, ranging between a mean of 144.5 ± 97.6 μS/cm for the rivers and 368.0 ± 125.9 μS/cm for the rock pools. Dams and drying stream habitats also recorded relatively high mean conductivity levels of between 269.8 ± 213.8 μS/cm and 290 ± 186.5 μS/cm, respectively. Measurements from drainages (168.5 ± 13.4 μS/cm), open sunlit puddles (168.8 ± 87.3 μS/cm), swamps (174.3 ± 59.2 μS/cm), dams (269.8 ± 213.8 μS/cm) and drying stream (290 ± 186.5 μS/cm) demonstrated marked variation in mean values. The lowest mean values were recorded in river habitats with measured mean ranges of 155.7 ± 88.4 μS/cm and 144.5 ± 97.6 μS/cm, respectively (Table 4). ANOVA test revealed a significant difference in electrical conductivity among the habitat types (ANOVA, n = 9, F = 7.1433, d.f.=8, 26, *p* < 0.01). Conductivity requirement range by both mosquito larva and predators varied markedly between 109.9 μS/cm to 396.2 μS/cm. However, ranges between 162.9 μS/cm and 166 μS/cm were most preferable based on mosquito larva and predator numbers captured in the shared habitats along the Mara River and its tributaries. Far fewer mosquito larvae and predators were present in samples at the extremes of these measurements.

Water pH measurements varied markedly between different habitats, ranging between 6.7 to 8.4. The highest mean value was recorded in open puddle habitats (8.2 ± 0.5), while the lowest (7.0 ± 1.3) was recorded in swamps. Drying stream and dam water pH measurements were comparable at 8.1 ± 0.6 and 8.1 ± 0.4, respectively. In drainages, the mean pH value was 7.3 ± 0.5, while river had mean of 7.3 ± 0.4. Rock pools, animal hoof prints and vegetated pools had mean pH values of 7.1 ± 0.8, 8.1 ± 0.3 and 8.0 ± 0.2, respectively (Table 4). There was significant differences in mean pH among the habitat types (ANOVA, n = 9, F = 9.443, d.f. = 8, 26, *p* < 0.01). Both mosquitoes and their predators were however abundant in the pH range of between 6.7 to 8.4, respectively.

Water temperature changes are influenced by many variables including time of sampling, source of the water and condition of the habitat. Along the Mara River, the highest mean temperature was recorded in the rock pools (26.2 ± 3.4°C), and animal hoof-prints (26.2 ± 1.9°C), followed by puddles (25.2 ± 2.3°C). River samples had the lowest temperature (19.7 ± 2.3°C). Temperatures in dam (24.4 ± 1.9°C), drainages (24.2 ± 0.7°C), swamps (23.2 ± 4.9°C) and drying stream (24.4 ± 1.9°C) varied slightly during the study period. Overall, the temperature variation within the Mara River and its tributaries as tested between July and August ranged from 18.0°C to 26.3°C. A significant difference in mean temperature was observed among the different habitat types (ANOVA, n = 9, F = 4.2004, d.f = 8, 26, *p* = 0.05). Both predator and prey preferred temperatures above 18°C. In particular, temperatures above 25°C contained the greatest number of both predator and prey. At these temperatures, some pool samplings recorded high predator numbers with very few prey, suggesting successful predation.

As presented in Table 4, the highest mean alkalinity (400 ± 282.8 mg/L) was recorded in the drainages while the lowest were recorded in dams and driver (100 ± 62.4 mg/L and 100 ± 99.2 mg/L), and vegetated pools (104 ± 73.0 mg/L). Similarly, variations between drying stream (126.2 ± 26.5 mg/L), puddles (104 ± 73.0 mg/L), rock pools (153 ± 60.8 mg/L), swamps (244.5 ± 274.6 mg/L) and animal hoof prints (133 ± 50.2 mg/L) were determined. Mean water alkalinity values differed significantly between habitat types along the Mara River (ANOVA, n = 9, F = 4.7042, d.f. = 8, 26, p < 0.001). Alkalinity range requirement for both mosquito larvae and predators in the shared habitats varied, with values ranging between 6.4 mg/L, and 406.1 mg/L. The most abundant collections of both larvae and their predators were in the range of 131.2 mg/L and 144.4 mg/L. From the above data, neither mosquito larvae nor predators had specific alkalinity requirement. Further analysis to determine the preferable alkalinity range requirement by both mosquito larvae and predators in the shared habitats indicated that only few insects preferred a range between 131.2 mg/L, and 144.4 mg/L, majority had a more wider requirement range. Similarly, this study found no specific preferences for hardness. For salinity, only swamps recorded slight salinity of 0.4 mg/L, while all the other sites recorded zero (Table 4).

**Figure 5:**
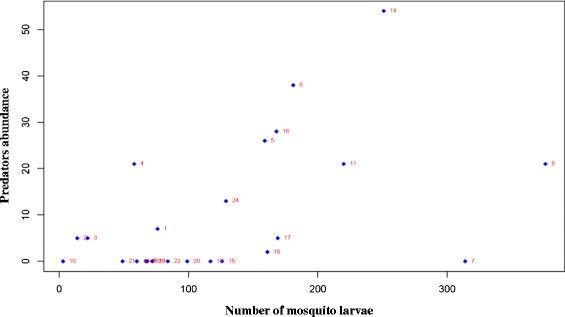
**Correlation matrix showing correlation between predators (blue stars) and the mosquitoes (absolute number) in shared habitat along the Mara river (n = 39).**

**Figure 6:**
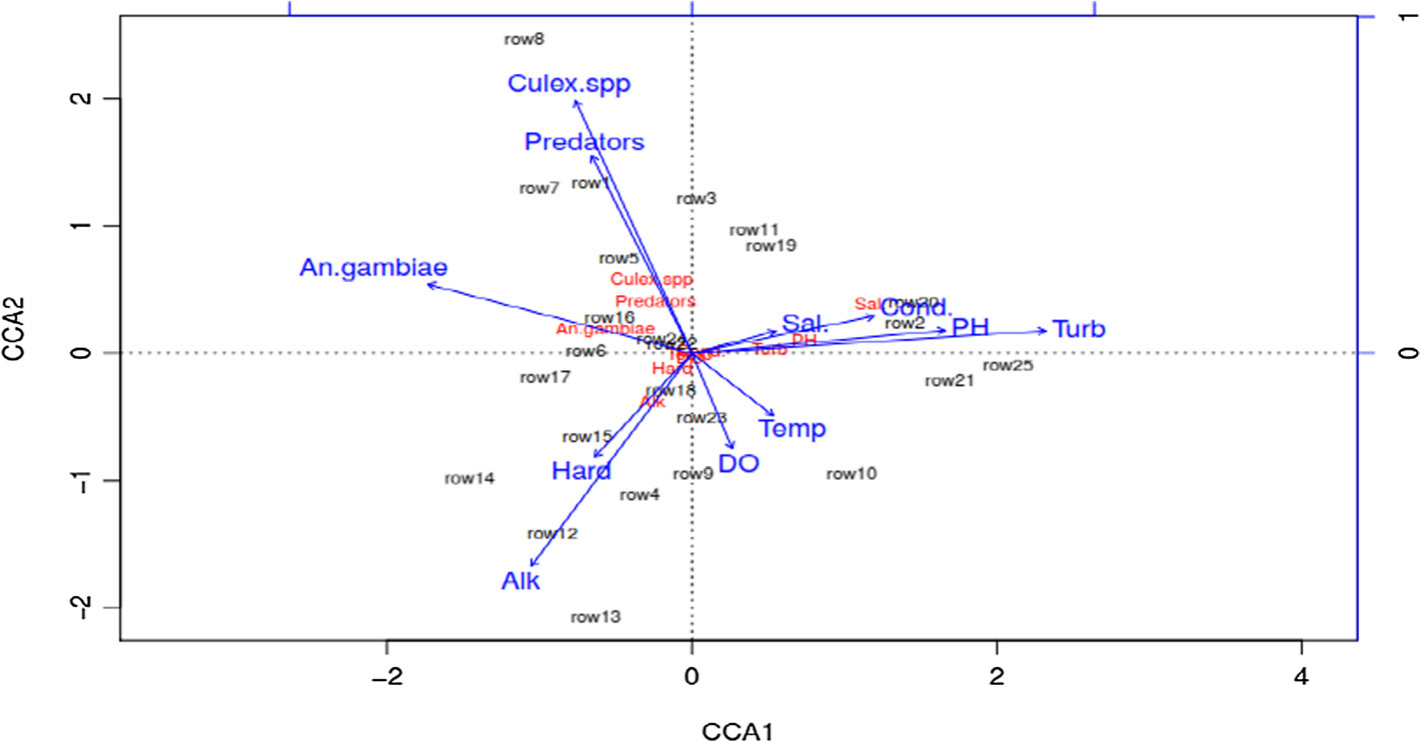
**Biplot of the overall effect of various environmental parameters recorded along the Mara river (n = 39).**

A negative binomial GLM results established that the abundance of the predators in habitats were partially driven by the presence of mosquito larvae (Z = 6.49, *p* < 0.001), and the prevailing water physico-chemical parameters (dissolved oxygen, Z = 3.34, *p* < 0.001; temperature, Z = 2.75, *p* < 0.001; and turbidity, Z =-3.65, *p* < 0.001), based on the best model with the smallest AIC (Table 5). To evaluate the strength and pattern of relationship between mosquito larvae and macroinverterbrate predators, a canonical correlation analysis was done. There was a strong correlation between the predators and mosquito larvae (R^2^=0.62, *p* < 0.005). Data from some sites showed inverse correlation between predators and prey (mosquito larvae), suggesting effective predation (Figure 5). The biplot (Figure 6), with intuitive interpretations of species-biotic interaction of all 9 variables confirmed that pH, alkalinity and hardness were less likely to influence mosquito larvae (*An. gambiae* complex and *Culex* spp.) and predators abundance. Dissolved oxygen and temperature were the most important factors that positively and directly correlated with both mosquito larvae and predators abundance based on quadrant reflection in the ordination analysis.

**Table 5:** **Final nb-GLM model for the response variable (mosquito larvae predators) and their predictors (mosquitoes and the physico-chemical parameters) that remained in the model, denoting factors influencing mosquito predators abundance in habitats along the Mara river**

**Variable**	**Estimate**	**Std. error**	**z value**	**Pr (>|z|)**
Intercept	-3.45	1.22	-2.83	0.005
Dissolved oxygen (DO)	0.38	0.11	3.34	<0.001
Temperature	0.07	0.03	2.75	0.006
Turbidity	-0.01	0.01	-3.63	<0.001
Mosquito larvae	0.41	0.10	6.49	<0.001

## Discussion

Malaria is a preventable and curable disease when encountered under ideal circumstances. However, under less ideal circumstances, or in regions where malaria is endemic such as the Sub-Saharan Africa, malaria morbidity and mortality continues to result in human and economic disability (WHO 2013). Resistance to chemical insecticides during the late 1950s resulted in an expected turn toward a search for biocontrol agents against the mosquito larvae. Some organisms are more chemical tolerant than others, and aquatic insects are sensitive to change of their environment. For instance, spraying of pesticides in agricultural fields along the river channel has been reported to have negative consequences on aquatic insects by Gereta et al. (2003). Therefore, alternative malaria control strategy of bio-environmental improvement techniques gives primary importance to anti-larval operations. Drying streams supported the greatest numbers of both mosquito larvae and predators during this sampling period and may be responsible for increasing natural predation in certain temporary habitats such as dams, open puddles and vegetated pools. This possibility is supported by the observation that certain ephemeral aquatic habitats had lower number of mosquitoes and higher predator abundance.

Water temperature, turbidity, and dissolved oxygen were found to be the main variables influencing the abundance and distribution of mosquito larva and predators in the aquatic habitats under investigation, even as test suggest that they opt more for clearer water. Within these habitats, ephemeral aquatic habitats had a diverse array of predators, which in some instances, correlated with negligible mosquito larvae numbers. These habitats results primarily due to human settlements. Brick making, cultivation, straying wildlife from the adjacent Masai Mara National Park and keeping of livestock have created animal hoof-prints. Consequently, open puddles, drainages and hoof-prints found in these areas supported a considerable number of mosquito and mosquito larvae predators.

The order Hemiptera were the most dominant and widespread representing 7 families. The 7 families were over-represented by Family Velidae and Genus Rhagovelia. Other predators of mosquito larvae belonged to the Order Odonata (which recorded 3 families dominated by family Coenagrionidae) and Order Coleoptera (which recorded 2 families dominated by Dytiscidae). The Hemipterans are regarded as effective predators of freshwater snails and mosquito larvae (Ohba and Nakasuji 2006). It is also well known that notonectids are voracious predators of mosquito larvae. Gilbert and Burns (1999) concluded that notonectid predators have the potential to alter mosquito communities via direct or indirect effects. Direct evidence of notonectid predation on mosquito larvae was later noted and this further confirmed their predominant role in mosquito larvae control (Chesson 1984).

The relatively low mosquito and predator numbers observed in the ephemeral habitats as compared to drying stream and swamps might have been due to several reasons. In addition to the fact that most of these habitats are open and might be accessible by the predators, earlier studies also reported that adult mosquitoes may have the ability to detect presence of predators and completely avoid ovipositing in such habitats, preferring instead to inhabit areas with swamps and grassy patches that can protect the immature stages (Vince et al. 1976; Nelson 1979; Coen et al. 1981; Heck and Thoman 1981; Stav et al. 1999). Previously, mosquitoes of the species *Culiseta longiareolata* were reported to detect chemicals from notonecta predators, and the instinct/cue can exist in the habitat for up to a week or more after their disappearance from the pool (Blaustein et al. 2004) and for *Culex* species, a period as low as two days have been reported (Blaustein et al. 2005).

We noted that the majority of predators were bonded to where there were lower densities of mosquito as reflected in graphical multi-correlation matrix. This supports the above aforementioned studies. However, higher number of predators and less prey could also be as a result of direct predation. It was therefore reasonable to expect fewer mosquitoes in habitats with higher number of predators. Other factors that have previously been reported to play an important role in habitat selection by various species of mosquitoes are volatile compounds produced by microbial population in the breeding sites (Sumba et al. 2008), chlorophyll content in the breeding sites (Munga et al. 2013) and other abiotic factors which can inhibit adult mosquitoes oviposition, coupled with habitat preferences (Minakawa et al. 1999, 2012).

The temperature recorded in the current study ranged between 18.0°C and 26.3°C, thus can be described as warm and are likely to support most of the predators especially the notonectids. Earlier studies showed that thermal conditions are especially important in predator–prey survival among aquatic organisms (Bailey 1989, Thomson 1978), especially those that are involved in size-dependent predation (Formanowicz 1986, Travis et al. 1985). However, while much research quantified in the physiological effects of temperature on specific organisms, few studies have been conducted to evaluate the effect of temperature on species interactions and their adaptive capacity to those ranges in field conditions.

Mosquito larvae and predators share the same habitats and thus establishing the role that pH plays in the regulation of colonization is critical. Both mosquito larvae and predators were not affected by pH in the final GLM model. This suggests that under the prevailing environmental conditions, both predators and mosquitoes could tolerate a wide range of pH. Further analysis to determine preferable pH range requirement by both mosquito larvae and predators established that values between 6.7 and 8.4 were tolerable, while values between 8.1 and 8.4 were most preferred, as evidenced by the highest number of both mosquito larvae and predators. The pH was largely basic in all habitat types. The adaptive range of pH by the insects was wide and within that range. Alkalinity levels were equally high ranging between 100 and 420 mg/L. This pH range has been reported as optimal for most aquatic biota including some mosquito predators. Most findings agree with the positive association of mosquito larvae and other aquatic insects under a wide range of pH values. For instance, Adebote et al. 2008 found mosquitoes of species of *An. ardensis*, *An. distinctus*, and *An. wilsoni* to be associated with pools of acidic nature (pH 5.86-6.55); however, *Cx. ingrami* occurred in partly acidic and partly alkaline pools (pH 5.86-9.85). Similarly, a study by Dejenie et al. (2011) on malaria vector control in Ethiopia established that almost all their study habitats were alkaline (pH >7) and both anopheline and culicine larvae were positively associated with this high (>7.0) pH. Our study thus is in agreement with the study of Dejenie et al. (2011), but does not support the findings of Adebote et al. (2008), which reported the preference of anopheline species to low pH values.

Along the Mara River, the mean turbidity was highest in rock pools, while the lowest level was recorded in swamps and drainages. The findings showed that turbidity levels across all sampled sites were exceedingly high. This scenario could be as a result of increased particulate matter such as clay, silt, organic matter, plankton and other microscopic organisms, which have been reported to interfere with the passage of light through water (American Public Health Association APHA 1998). The increased particulate matter could have been contributed by anthropogenic activities such as deforestation, river bank cultivation, soil erosion (due to overgrazing among others), all occurring in the watershed. In addition, urbanization facilitates transportation of waste into the river channel through increased run-offs, while livestock trampling effect at watering points and along the river banks also contributes significantly to high turbidity levels of surface waters. All these activities can create suitable habitats for mosquitoes as was previously reported by Matthys et al. (2006).

A habitable aquatic ecosystem requires a good supply of dissolved oxygen in the water system (Davis 1975). Along the Mara River basin, the mean dissolved oxygen was highest in the river followed by rock pools, while the lowest was recorded in swamps. A significant difference in mean dissolved oxygen was observed among the different habitat types. Faster flowing sections of rivers and drying stream and sections that flow through riffles or small waterfalls have better oxygenated waters than slow flowing sections of rivers or rivers that have been modified as straight channels. Dissolved oxygen concentration in water is dependent on physical, chemical, biological and microbiological processes. Low dissolved oxygen concentrations (<3 mg/L) in fresh water ecosystems are indicative of high pollution levels (Okbah and Tayel 1999). However, in the current study, some aquatic habitats recorded dissolved oxygen levels insufficient to support aquatic life. Analysis to determine preferable level of dissolved oxygen range required by both mosquito larvae and predators in the shared habitat indicated that values ranging between 6.0 mg/L, and 6.5 mg/L were most preferred. However, some mosquito larvae were found in water samples with dissolved oxygen concentration as low as 2.3 mg/L. The most common cause of low oxygen levels is the off-load of organic material into the water system (such as agricultural run-offs). Nevertheless, more mosquito larvae were collected in slow-flowing drying stream and swamps where the mean oxygen was relatively low. The majority were mainly *Culex* spp., however *Anopheles* species were also higher as compared to the ephemeral habitats. previous studies reported *Culex* spp. to occur in habitats with wide range of dissolved oxygen (DO) levels (Opoku et al. 2007).This may also suggest that majority of predators were unable to survive in polluted water or the water volume was sufficient to maintain high number of mosquitoes beyond predator’s capacity. Also, streams and rivers have burrows at the banks which can help in refuge of the mosquitoes. In fact, majority of the mosquitoes were captured at the edges of the streams, most of which are vegetated.

The majority of *Anopheles* and *Culex* spp. larvae were found inhabiting pools adjacent to the Mara River created by receding river waters, some of which had relatively high dissolved oxygen levels. These findings are consistent with those of Dejenie et al. (2011) who also reported that both Anopheline and Culicine larvae were positively associated with dissolved oxygen. Studies by Muturi et al. (2008) also indicated similar association of *Anopheles* spp. larvae and other mosquito larvae with dissolved oxygen. Likewise, Oyewole et al. (2009) emphasized that optimum dissolved oxygen is superlative to the survival of the *Anopheles* larvae.

Water hardness is usually a result of the presence of multivalent metal from minerals dissolved in water. In the aquatic environment, ions result from abundance of calcium and magnesium in water. The highest mean hardness was recorded in the drainages, while the lowest were recorded in dams and swamps. A correlation matrix established that there was a positive correlation between mosquito larvae and predators in the presence of hardness. However, a negative correlation was observed between hardness and predators in the shared habitats suggesting that most predators require lower water hardness levels to survive. Analysis to determine the preferable level of hardness range requirement by both mosquito larvae and predators in the shared habitats indicated that values as wide as 58.5 mg/L to 397.1 mg/L, were favorable. The wide range of water hardness observed could be due to differences in buffering capacity of the waters across habitat types, as hardness values are not consistent across the basin. Elevated values in some areas could be as a result of sewer supply from the nearby towns or spills of fertilizer from the nearby farms. Other established sources could be the local geology (Lawrence 2007). However, few insects showed preference for specific hardness values. It was also of interest to note that along the Mara River, most aquatic habitats had meagre detectable level of salinity. Only swamps recorded salinity level of 0.4 mg/L. However, the influence of salinity along the Mara River could not be statistically evaluated as a result of insufficient sample numbers.

In the current study, rock pools, dams and drying stream recorded the highest mean conductivity, while swamps and drainages had the lowest conductivity values. For both mosquito larvae and predators, a perfect linear requirement with conductivity in the same habitat was demonstrated within the ranges of between 162.9 μS/cm to166μS/cm by both mosquito larvae and predator residing in the same habitats. The high levels were due to elevated dissolved solids and contaminants especially electrolytes. Potential sources of these contaminants are destruction of the forest cover (which in the process, increase the litters) and human activities experienced along the river channel (that creates drainages and pools suitable for mosquito breeding). Mati et al. (2008) and Jordao et al. (2007) reported increased destruction of the upper catchment of the Mau forest and elevated level of pollution, attributable to high levels of waste water discharged into the river from different origins.

Previously, dissolved oxygen, temperature and conductivity were reported to positively correlate with macroinvertebrate community structure as a whole (Spieles and Mitsch 2000). In the current study, no direct relationship was detected between conductivity and predator abundance in the GLM model. However, there was a limited range of conductivity requirement levels preferable to both mosquito and predator population. The conductivity of a river or stream should remain within a specified range to allow for a successful biologically functional system. Changes in conductivity are often used as water pollution indicator. Urban run-offs and industrial pollution are often characterized by high conductivity.

Ordination analysis, factoring in all the variables showed that dissolved oxygen and temperature had direct influence on mosquito larvae and predator abundance, while other biotic factors indicated meagre, opposite or insignificant role, supporting the earlier notion that within aquatic habitat, both macroinvertebrates and mosquitoes can be sensitive to factors affecting water quality. Gauch (1982) concur that ordination primarily endeavors to represent sample and species relationships as faithfully as possible in order to choose precisely which tool is necessary for immediate use. Predator abundance was strongly positively correlated with the increasing number of mosquitoes, suggesting that carefully selected predators may play a noble role in controlling mosquitoes as compared to the water physico-chemical parameters. Specifically, the abundance of predators, *Culex* and *Anopheles* spp. were perfectly correlated in ordination analysis. pH, turbidity, conductivity, alkalinity, hardness and salinity, whereas some were correlated with each other, the higher they were, the less likely that the mosquito and predators would inhabit, except with the availability appropriate water prysico-chemical requirement range. Previous studies reported that thermal pollution, pesticides and organic compounds affects the water physico-chemical parameters, thus interfering with aquatic invertebrate diversity and composition (Hilsenhoff 1988). This may also partially explain the abundance of Hemiptera, as compared to the other two aquatic insect orders; Odonata and Coleoptera. Most of the insects in the Order Hemiptera have been acclaimed as pollution-tolerant (Joshi 2012), and their population was found to be higher than any other order along the Mara River. Other known sensitive taxa such as Plecoptera were completely absent from all the sites, suggesting that the waters might have been polluted.

Downstream sites of the Mara River had greater flow rates, more stable physico-chemical parameters, and potentially greater influence from tributaries in the biotic interactions. Of interest is the fact that more fish species were present in middle and lower Mara River sites as compared to upstream sites, where most physic-chemicals had extreme ranges, and thus the biotic interactions were potentially more important in regulating fish abundance upstream. We speculate that physico-chemical variables are important influencers on fish diversity and abundance in the Mara River. Therefore, future studies determining fish community structure and the role of the biotic factors should further elucidate their importance with proper design and robust analysis.

## Conclusions

Mosquito resistance to chemical insecticides is a growing problem, and increasing attention is being paid to alternative control methods. The findings reported herein provide new information on the presence of macroinvertebrate and mosquito larvae within the Mara River and its tributaries. Some of these predatory species have been evaluated as bio-control agents in the worldwide campaign to control malaria vectors. This study also defines the most preferable physico-chemical parameter range dependency by the predators and mosquito larvae. Understanding abiotic and biotic factors which favour mosquitoes and macroinveterbrate co-occurrence, may contribute to the control of malaria.

## Limitations of the study

This study was designed and conducted during the dry period along the Mara River tributaries and the main Mara River in Kenya and Tanzania. We presumed that samples collected during this dry period would represent extremes within this ecosystem. From our data extremes, multiple variables were measured. However, we recognize the limitations of a crossectional study in which all aspects of habitat parameters, especially changes in the physico-chemical parameters over time are not represented. Also, a more extensive fish sampling should have been conducted and in a more scientific manner that would ensure concrete conclusion are made, including generalization of the results to larger groups.

## Abbreviation

GLM: Generalized linear model
